# Diversity of Capsular Polysaccharide Gene Clusters in Kpc-Producing *Klebsiella pneumoniae* Clinical Isolates of Sequence Type 258 Involved in the Italian Epidemic

**DOI:** 10.1371/journal.pone.0096827

**Published:** 2014-05-13

**Authors:** Marco Maria D’Andrea, Francesco Amisano, Tommaso Giani, Viola Conte, Nagaia Ciacci, Simone Ambretti, Luisa Santoriello, Gian Maria Rossolini

**Affiliations:** 1 Department of Medical Biotechnologies, University of Siena, Siena, Italy; 2 Operative Unit of Clinical Microbiology, St. Orsola-Malpighi University Hospital, Bologna, Italy; 3 Unit of Microbiology, “S. Corona” Hospital, Pietra Ligure, Italy; 4 Department of Experimental and Clinical Medicine, University of Florence, Florence, Italy; 5 Clinical Microbiology and Virology Unit, Florence Careggi University Hospital, Florence, Italy; Inserm U914 and South Paris University, France

## Abstract

Strains of *Klebsiella pneumoniae* producing KPC-type beta-lactamases (KPC-Kp) are broadly disseminating worldwide and constitute a major healthcare threat given their extensively drug resistant phenotypes and ability to rapidly disseminate in healthcare settings. In this work we report on the characterization of two different capsular polysaccharide (CPS) gene clusters, named *cps*_BO-4_ and *cps*_207-2_, from two KPC-Kp clinical strains from Italy belonging in sequence type (ST) 258, which is one of the most successful ST of KPC-Kp spreading worldwide. While *cps*_BO-4_ was different from known 78 K-types according to the recently proposed typing schemes based on the *wzi* or *wzc* gene sequences, *cps*_207-2_ was classified as K41 by one of these methods. Bioinformatic analysis revealed that they were represented in the genomic sequences of KPC-Kp from strains of ST258 from different countries, and *cps*_BO-4_ was also detected in a KPC-Kp strain of ST442 from Brazil. Investigation of a collection of 46 ST258 and ST512 (a single locus variant of ST258) clinical strains representative of the recent Italian epidemic of KPC-Kp by means of a multiplex PCR typing approach revealed that *cps*_BO-4_ was the most prevalent type, being detected both in ST258 and ST512 strains with a countrywide distribution, while *cps*_207-2_ was only detected in ST258 strains with a more restricted distribution.

## Introduction

The capsular polysaccharide (CPS or K-antigen) is a recognized virulence factor of *Klebsiella pneumoniae* [1], [2]. This component exhibits a remarkable intra-specific structural diversity which translates into different antigenic properties that may be relevant to bacterial virulence [2]–[4].

CPS diversity has classically been detected by serotyping techniques [5], but genotyping systems have recently been developed, offering several advantages *vs.* the conventional serotyping approach [6]–[10]. Among systems that do not require a sequencing step, a PCR-based typing system has been proposed for the detection of isolates of the K1, K2, K5, K20, K54 and K57 capsular types, that are commonly associated with invasive diseases or having a prominent pathogenicity [6]. Conversely, two systems based on amplification and sequencing of the conserved *wzi* and *wzc* genes were recently proposed to determine the K-type of *K. pneumoniae* [9], [10].

During the last years, strains of *K. pneumoniae* producing KPC-type carbapenemases (KPC-Kp) belonging in sequence type (ST) 258 and related variants (e. g. ST512, ST437 and ST11) have undergone a global dissemination, with epidemic diffusion in some areas of North and South America, Europe and Asia [11]–[18]. Infections caused by these strains pose a major challenge due to their extended antibiotic resistance phenotypes and ability to rapidly disseminate in healthcare settings, and are associated with high mortality rates [19]–[20]. Detailed knowledge on the CPSs of these strains, however, is still limited. A ST258 KPC-Kp strain from Greece has recently been reported to express a K41 serotype CPS [21], while the chemical structure of the CPS of two representatives of an outbreak clone of ST258 KPC-Kp from USA has recently been described [22].

In this work we have characterized two different *cps* gene clusters from two KPC-Kp clinical strains of ST258 from Italy, and report on their distribution in a collection of KPC-Kp isolates of ST258 and ST512 representative of the recent Italian epidemic. We also propose a modification to a previously established PCR-based CPS typing system [6], to include recognition of these CPS types.

## Results

### Characterization of Two Different CPS Gene Clusters in ST258 KPC-Kp Strains of Clinical Origin

The CPS gene cluster of two KPC-Kp strains of clinical origin, KKBO-4 and KK207-2, were characterized by an HTGS approach. The two strains had been isolated in 2010 from bloodstream infections of inpatients in two different Italian hospitals and produced either KPC-2 (KK207-2) or KPC-3 (KKBO-4). They were both of ST258, and exhibited a related although not identical XbaI PFGE profile [12] (difference of two bands, data not shown).

Comparison of the draft genomes using GGDC 2.0 confirmed the close relatedness between the two strains at the genomic level (intergenomic distance of 0.0015). Despite this close relatedness, however, the *cps* gene clusters of the two strains were significantly different from each other.

**Figure 1:**
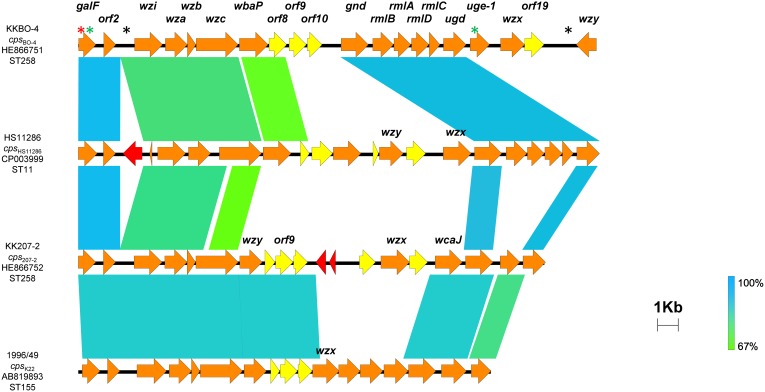
Comparison of the CPS gene clusters from *K. pneumoniae* strains KKBO-4 (*cps*_BO-4_), HS11286 (*cps*_HS11286_), KK207-2 (*cps*_207-2_), and 1996/49 (K-type 22, *cps*_K22_). Sequence accession numbers and STs for the respective strains are also indicated (the ST of strain 1996/49 was deduced from ref. 30). The CPS gene cluster of strain 8238 (K-type 37) (accession number AB819894), differing from *cps*_K22_ by a single nucleotide deletion resulting in a frameshift mutation located in a putative acetyltransferase downstream *gnd*, is not included for simplicity. Homologous regions are connected by areas of different colors reflecting the degree of nucleotide identity (from 67% to 100%). Open reading frames encoding transposases are colored in red, while those encoding hypothetical glycosyltransferases are colored in yellow. The locations of synonymous, non-synonymous and intergenic single nucleotide variations (SNVs) occurring between the CPS gene clusters of KKBO-4 and Kp13 are indicated by green, red and black stars, respectively. The *cps*_207-2_ gene cluster exhibited regions of similarity to *cps*_BO-4_ including the conserved *galF*-*wzc* region (83.2% of nucleotide identity), and the conserved *gnd* and *ugd* genes (95.5% and 96.8% nucleotide identity, respectively).

The CPS gene cluster of KKBO-4 (named *cps*_BO-4_) was found to be 26,587 bp-long, consisting of 20 ORFs (from *galF* to *wzy*), and was characterized by the presence of the K-antigen flippase- and polymerase-encoding genes (*wzx* and *wzy*, respectively) at the 3′-end, and by the presence of the *rmlBADC* operon for the synthesis of dTDP-L-rhamnose in the central region (Fig. 1).

The *cps*_BO-4_ gene cluster was identical or very similar to those present in a number of ST258 *K. pneumoniae* strains from different countries, whose genome sequences are available in the public domain, and also very similar to that previously described in a ST442 KPC-Kp strain (Kp13) that caused an outbreak in Brazil [23] (Table 1 and Fig. 1). Compared to the CPS gene cluster of *K. pneumoniae* HS11286 (ST11, a single locus variant of ST258) [29], *cps*_BO-4_ exhibited significant similarities in some regions (e. g. from *galF* to *orf8* and from *gnd* to *uge-1*, comprising the *rmlBADC* operon), but also substantial differences in the central and the 3′-region of the gene cluster (Fig. 1).

**Table 1:** Differences between the CPS gene clusters of strains KKBO-4 (*cps*_BO-4_, 26,587 bp) or KK207-2 (*cps*_207-2_, 23,994 bp) and closely related CPS gene clusters detected in other sequenced genomes.

Strains	STs	Countries	*cps*-types	NucleotideDifferences	Gene Mutations(AminoAcid Differences)	References/Accession numbers
KPNIH21^a^	258	USA	*cps*_BO-4_	-	-	[24]
ST258–490	258	Israel	*cps*_BO-4_	-	*-*	[25]
ST258 K26BO^b^	258	Italy	*cps*_BO-4_	-	*-*	[26]
MP14	258	South Korea	*cps*_BO-4_	-	*-*	[27]
ATCC BAA-1705	258	USA	*cps*_BO-4_	1	*galF* a191c (E64A)	[28]
UHKPC02^c^	258	USA	*cps*_BO-4_	1	*galF* a191c (E64A)	ARSK00000000.1
BIDMC 12C	258	USA	*cps*_BO-4_	2	*galF* a191c (E64A)	AXLG00000000.1
					*gnd* t1220c (V407A)	
					*galF* a191c (E64A)	
Kp13	442	Brazil	*cps*_BO-4_	5^d^	*galF* t366a (silent)	[23]
					*uge-1* g150a (silent)	
UHKPC06^c^	258	USA	*cps*_207-2_	1	*wzc* c1936a (P646T)	ARSJ00000000.1

The dash indicates 100% identity. The cut-off values used for the inclusion in the analysis were ≥99% nucleotide identity and ≥99% of query coverage, based on results from a BLAST search performed at the NCBI website (http://blast.ncbi.nlm.nih.gov/) using either nr or wgs databases, using default values but without the low complexity filter option.

a KPNIH21 was chosen as a representative of the outbreak clone described in reference 24.

b strains ST258 K26BO and ST258 K28BO, both described in reference 26, were characterized by identical CPS gene clusters.

c strains UHKPC02 and UHKPC06 were representatives of those included in the *Klebsiella pneumoniae* Genome Sequencing Center Project (http://gsc.jcvi.org/projects/gsc/klebsiella_pneumoniae/index.php).

d 2 out of 5 SNVs are located in intergenic regions.

According to the *cps*-typing protocol based on sequencing of the *wzi* gene [9] *cps*_BO-4_ showed a single nucleotide difference with the wzi81-K81 reference amplicon. According to the *cps*-typing protocol based on sequencing of the *wzc* gene [10] *cps*_BO-4_ was <80% identical to that of any other reference sequence.

The CPS gene cluster of strain KK207-2 (named *cps*_207-2_) was found to be 23,994 bp-long, consisting of 19 ORFs (from *galF* to *ugd*). It did not contain the *rmlBADC* operon but contained original genes, of which some encode putative glycosyltransferases, located between the *wzy* and *wcaJ* genes (Fig. 1). The *cps*_207-2_ gene cluster was very similar to those present in ST258 *K. pneumoniae* strains from USA, whose genome sequences are available in the public domain (Table 1). It also exhibited regions of similarity with the CPS gene clusters of *K. pneumoniae* strains 1996/49 and 8238, producing CPS of the K22 and K37 serotype, respectively, and with both *cps*_BO-4_ and *cps*_HS11286_ (Fig. 1).

According to the *cps*-typing protocol based on sequencing of the *wzi* gene [9] *cps*_207-2_ was identical to the wzi29-K41 reference amplicon. According to the *cps*-typing protocol based on sequencing of the *wzc* gene [10] *cps*_207-2_ was 93% identical to the K22_ref and K37_ref reference sequences.

Taken together, these results suggested that the CPS composition of KK207-2 was different from that of KKBO-4, demonstrating that at least two different types of CPS gene clusters may be found in KPC-Kp of ST258, and that *cps*_BO-4_-like gene clusters can also be found in KPC-Kp of unrelated STs such as ST442.

### Analysis of the *cps* Gene Clusters in a Contemporary Collection of ST258 and ST512 KPC-Kp Strains from the Italian Epidemic

**Figure 2:**
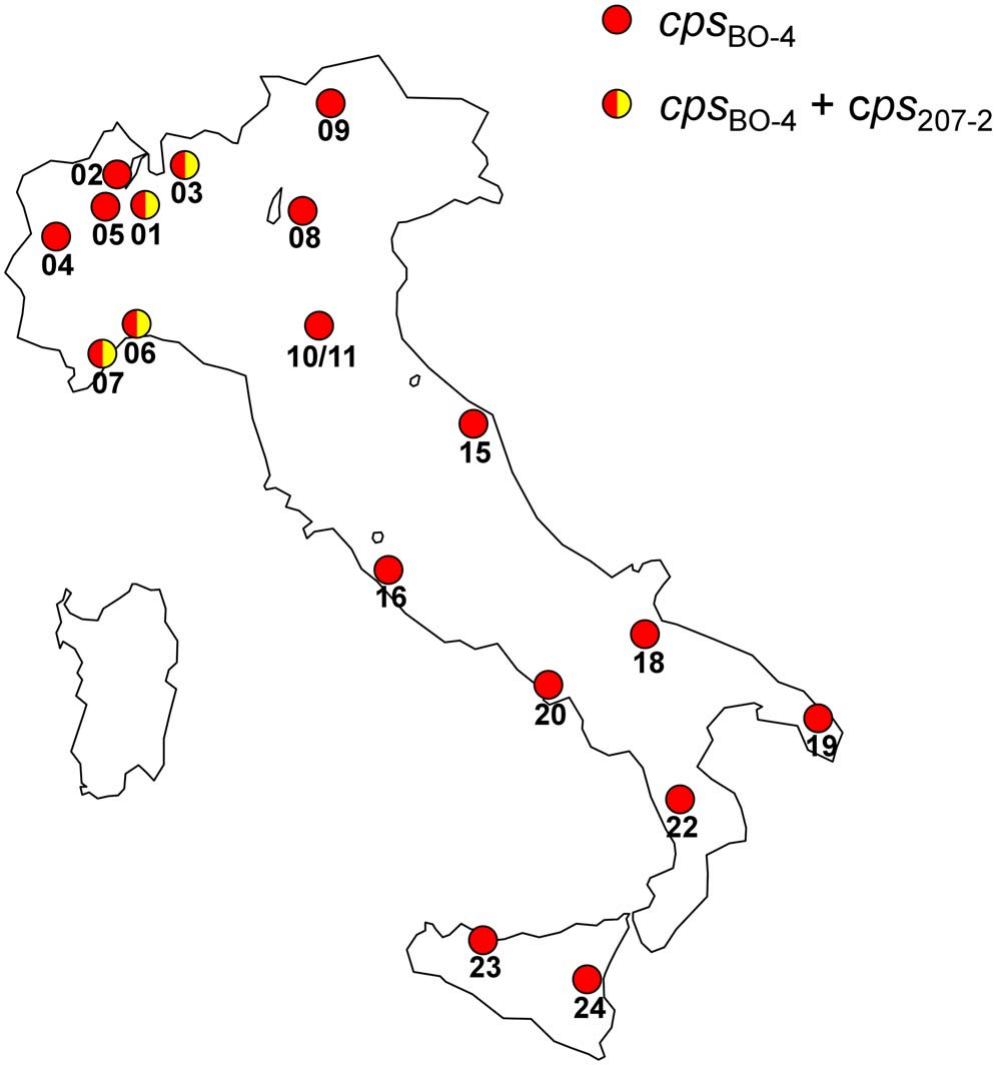
Map showing the distribution of Italian centers from which the 46 KPC-Kp strains of ST258 or ST512 investigated for CPS typing by the modified multiplex PCR were originated, and distribution of the different types of *cps* gene clusters. Centers were as follows: 01, Milan; 02, Varese; 03, Lecco; 04, Torino; 05, Novara; 06, Genoa; 07, Sanremo; 08, Verona; 09, Bolzano; 10, Modena; 11, Modena; 15, Ancona; 16, Rome; 18, Foggia; 19, Lecce; 20, Naples; 22, Cosenza; 23, Palermo; 24, Catania.

A multiplex PCR protocol derived from that originally proposed by Turton *et al*. [6], modified to detect the *cps*_BO-4_ and *cps*_207-2_ gene clusters, was used to analyze a collection of 46 nonreplicate KPC-Kp clinical strains of ST258 or ST512 isolated from 19 different centers (Fig. 2) during the first Italian countrywide survey on carbapenem-resistant *Enterobacteriaceae* [12] and selected as representatives of the recent Italian KPC-Kp epidemic. Nine additional carbapenem-resistant but KPC-negative *K. pneumoniae* strains with different carbapenem-resistance mechanisms (production of VIM-1, of OXA-48, or of an extended-spectrum beta-lactamase in presence of a permeability defect), isolated during the same survey, were also analyzed for comparison.

Of the 46 KPC-Kp strains, 38 (82.6%) carried a *cps*_BO-4_-like cluster, while the remaining 8 (17.4%) carried a *cps*_207-2_-like cluster (Table 2). The *cps*_BO-4_-like gene cluster was detected in both ST258 and ST512 strains from all 19 centers, while the *cps*_207-2_-like gene cluster was only detected in ST258 strains from 4 centers (Table 2, Fig. 2). The 9 KPC-negative strains were not typeable by the modified multiplex PCR, with the exception of one isolate identified as K2, indicating that none of those strains carried *cps*_BO-4_-like or *cps*_207-2_-like gene clusters. According to the *wzi* sequence-based typing method [9], the isolates were genotyped as K2 (n = 1), K9 (n = 1), K17 (n = 3), K38 (n = 1), K14/64 (n = 1), K15/17/50/51/52 (n = 2). Results were consistent with the fact that none of these K-types, except K2, could be detected by the modified multiplex PCR method.

**Table 2:** KPC-Kp strains of clinical origin from the Italian nationwide survey investigated for the nature of the *cps* gene cluster by the modified multiplex PCR developed in this work.

Strain ID	Sample	PFGE	ST	*cps*-type
01C03	Urine	A6	258	*cps*_207-2_
01C06	Urine	A3	258	*cps*_207-2_
01C08	Wound exudate	A6	258	*cps*_207-2_
01C22	Wound exudate	A0	512	*cps*_BO-4_
02C01	Urine	A5	512	*cps*_BO-4_
02C06	Wound exudate	A2	258	*cps*_BO-4_
03C06	Wound exudate	A4	512	*cps*_BO-4_
03C08	Urine	A3	258	*cps*_207-2_
03C12	Urine	A1	258	*cps*_207-2_
04C35	Urine	A0	512	*cps*_BO-4_
04C38	Bronchial aspirate	A4	512	*cps*_BO-4_
04C49	Bronchial aspirate	A2	258	*cps*_BO-4_
05C15	Blood	A4	512	*cps*_BO-4_
06C02	Urine	A3	258	*cps*_207-2_
06C04	Urine	A4	512	*cps*_BO-4_
06C05	Urine	A2	258	*cps*_BO-4_
06C07	Bronchial aspirate	A3	258	*cps*_207-2_
06C19	Blood	A0	512	*cps*_BO-4_
07C06	Urine	A3	258	*cps*_BO-4_
07C07	Bronchial aspirate	A2	258	*cps*_207-2_
08C02	Urine	A0	512	*cps*_BO-4_
08C04	Urine	A2	258	*cps*_BO-4_
09C06	Urine	A1	258	*cps*_BO-4_
10C04	Urine	A5	512	*cps*_BO-4_
10C09	Wound exudate	A4	512	*cps*_BO-4_
11C07	Urine	A5	512	*cps*_BO-4_
15C05	Bronchial aspirate	A4	512	*cps*_BO-4_
15C10	Urine	A2	258	*cps*_BO-4_
15C15	Bronchial aspirate	A0	512	*cps*_BO-4_
15C18	Urine	A1	258	*cps*_BO-4_
16C05	Blood	A4	512	*cps*_BO-4_
16C12	Wound exudate	A2	258	*cps*_BO-4_
18C01	Bronchial aspirate	A4	512	*cps*_BO-4_
18C22	Abscess	A2	258	*cps*_BO-4_
18C24	Wound exudate	A1	258	*cps*_BO-4_
19C09	Blood	A5	512	*cps*_BO-4_
19C11	Blood	A2	258	*cps*_BO-4_
20C14	Blood	A6	258	*cps*_BO-4_
22C06	Bronchial aspirate	A2	258	*cps*_BO-4_
22C09	Urine	A1	258	*cps*_BO-4_
22C24	Wound exudate	A4	512	*cps*_BO-4_
23C10	Urine	A1	258	*cps*_BO-4_
23C13	Bile	A6	258	*cps*_BO-4_
24C02	Blood	A2	258	*cps*_BO-4_
24C20	Bronchial aspirate	A2	258	*cps*_BO-4_
24C21	Bronchial aspirate	A2	258	*cps*_BO-4_

The first two characters of each strain ID identify the center from which the isolate was obtained. Identifiers are as reported in the legend to Fig. 2.

## Discussion

Results of this work showed that KPC-Kp belonging to ST258, which have largely contributed to the epidemic dissemination of the KPC-type beta-lactamases in Italy and elsewhere [12], [20], can be equipped with at least two different types of CPS gene clusters, here named *cps*_BO-4_ and *cps*_207-2_. The former type was more prevalent in a collection of representative isolates from the recent Italian epidemic, being also present in strains of ST512. The differences in the nature of these CPS gene clusters could be related with differences in the ability of spreading and virulence of different clones, which will deserve further investigation.

The detailed chemical structure of *cps*_BO-4_ was recently solved for two representatives of the outbreak clone of KPC-Kp found at the Clinical Center of the U.S. National Institutes of Health [22], [24]. The Authors demonstrated that this CPS type is structurally different from any other published *K. pneumoniae* CPS, even if similarity to *K. pneumonia*e K19 and K34 antigens was observed, possibly explaining the cross-reactivity of this CPS with the K34 antiserum [22]. These results corroborate the hypothesis that *cps*_BO-4_-like gene clusters belong to a novel capsular type, as also suggested by the results obtained for *cps*_BO-4_ using the typing methods based on *wzi* and *wzc* gene sequences.

On the other hand, results obtained for *cps*_207-2_ using the above genotyping methods were not in agreement between each other. In fact, while according to the *wzc*-based method [10] *cps*_207-2_ corresponded to a new K-type, according to the *wzi-*based method [9] this gene cluster corresponds to the known K41 K-type. The result obtained with the *wzi*-based method could be consistent with the finding that a strain of KPC-Kp of ST258, representative of the dominant clone circulating in Greece during 2009–2011, was serotyped as K41 [21]. This finding also suggests that this K-type has achieved a significant distribution in some settings, and it would therefore be interesting to further investigate the nature of the whole CPS gene cluster in KPC-Kp strains of K-type 41.

Data presented here also confirmed that the CPS gene cluster do not unambiguously correlate with any particular ST, confirming the notion that CPS gene clusters can be exchanged between different strains of *Enterobacteriaceae* species [30]–[32].

## Materials and Methods

### Bacterial Strains

Two KPC-Kp strains of ST258, KKBO-4 and KK207-2, isolated in 2010 from two different Italian hospitals and epidemiologically unrelated with each other, were used for high-throughput genome sequencing (HTGS) analysis and characterization of their *cps* gene clusters.

Forty-six additional KPC-Kp strains of ST258 or ST512 plus nine carbapenem-resistant but KPC-negative *K. pneumoniae* strains of different STs were investigated by the modified multiplex PCR for CPS genotyping developed in this work. These strains were selected as representative of the recent Italian epidemic of carbapenem-resistant *K. pneumoniae* from a collection of clinical isolates obtained during the first nationwide survey on carbapenem-resistant *Enterobacteriaceae* carried out in Italy in 2011 [12].

### High-Throughput Genome Sequencing and Analysis of Sequence Data

HTGS was performed using a HiSeq 2000 Illumina platform and a paired-ends protocol with an average insert size of 300 bp. Reads were assembled using ABySS [33]. GGDC software was used to assess the genomic diversity of the investigated isolates [34]. HTGS for strain KKBO-4 has been described previously [35]. The web interface of BLAST available at the NCBI website was used to compare the CPS gene clusters of the two strains with homologues in the nr or wgs databases [36]. CPS gene clusters sequences were aligned with ClustalX [37]. Structural comparisons of KKBO-4, KK207-2 and other published *cps* gene clusters were performed with EasyFig. [38].

The nucleotide sequences of the *cps* gene clusters of KKBO-4 and KK207-2 were deposited in the DDBJ/EMBL/GenBank databases under accession numbers HE866751 and HE866752, respectively.

### Multiplex PCR for CPS Typing

CPS gene clusters were genotyped using a multiplex PCR approach as previously described [6], modified by including two additional primer pairs designed to amplify specific targets in the *cps* gene clusters described in this paper: wziBO-4F (5′-CGGTTTCCTGATGCAGCGG-3′) and wziBO-4R (5′-ATCATGTGCTTCCAGGTACC-3′), targeting the *wzi* gene of the *cps*_BO-4_ gene cluster, and hgt207-2F (5′-GCAGCTGATTCCAGAAATATTG-3′) and hgt207-2R (5′-CATATGCTCTAATACCAAAGCC-3′), targeting a hypothetical glycosyltransferase gene of the *cps*_207-2_ gene cluster (*orf9* in Fig. 1). These two additional primer pairs yielded amplicons of 478 and 352 bp, respectively, being suitable for the inclusion in the multiplex PCR because of the unique band sizes. Primers K.pneumoniae Pf and K.pneumoniae Pr1, designed for the identification at the species level of *K. pneumoniae* and included in the original multiplex PCR protocol, were not included in the reaction mix.

### Addendum in Proof

After the revised version of this manuscript had been submitted, two articles have been published reporting the occurrence of two distinct *cps* gene clusters in *Klebsiella pneumoniae* isolates belonging to the ST258 clonal lineage [39], and the development of a PCR-based assay for their detection [40]. The two gene clusters, named *cps-1* and *cps-2*, correspond to *cps*_207-2_ and *cps*_BO-4_ described here, respectively, while the PCR assay targets the different *wzy* genes of the two clusters. At the same time, an additional article has been published reporting that *K. pneumoniae* isolates of ST258 are characterized by *cps* gene clusters carrying a novel *wzi* allele (*wzi*-154) [41], that is identical to the *wzi* allelic variant of *cps*_BO-4_.
